# Understanding adherence to the recording of ecological momentary assessments in the example of tinnitus monitoring

**DOI:** 10.1038/s41598-020-79527-0

**Published:** 2020-12-31

**Authors:** Miro Schleicher, Vishnu Unnikrishnan, Patrick Neff, Jorge Simoes, Thomas Probst, Rüdiger Pryss, Winfried Schlee, Myra Spiliopoulou

**Affiliations:** 1 Institute of Technical and Business Information Systems, Otto-von-Guericke-University Magdeburg, Magdeburg, Germany; 2 Department of Psychiatry and Psychotherapy of Regensburg University, Regensburg, Germany; 3 University Research Priority Program ‘Dynamics of Healthy Aging’, University of Zurich, Zurich, Switzerland; 4 Department for Psychotherapy and Biopsychosocial Health, Danube University Krems, Krems, Austria; 5 Institute of Clinical Epidemiology and Biometry, University of Würzburg, Würzburg, Germany

**Keywords:** Patient education, Quality of life

## Abstract

The recording of Ecological Momentary Assessments (EMA) can assist people with chronic diseases in monitoring their health state. However, many users quickly lose interest in their respective EMA platforms. Therefore, we studied the adherence of users of the mHealth app TrackYourTinnitus (TYT). The app is used to record EMA in people with tinnitus. 1292 users, who interacted with the app between April 2014 and February 2017, were analyzed in this work. We defined “adherence” based on the dimensions of interaction duration and interaction continuity. We propose methods that are able to predict the (dis)continuation of interaction with the app and identify user segments that are characterized by similar patterns of adherence. For the prediction task we used the data of the questionnaires MiniTF and TSCHQ, which are filled in when the users enter TYT for the first time. Additionally, time series of the eight items of the daily EMA questionnaire were used. The distribution of user activity pertaining to the adherence dimension of interaction duration revealed a very skewed distribution, with most users giving up after only 1 day of interaction. However, many users returned after interrupting for some time. Some of the MiniTF items indicated that the worries of users might have lead to an increased likelihood of returning back to the app. The MiniTF score itself was not predictive, though. The answers to the TSCHQ items, in turn, pointed to user strata (more than 65 years of age at registration), which tended towards higher interaction continuity. As the registration questionnaires predicted adherence only to a limited extent, it is promising to study the activities of the users in the very first days of interaction more deeply. It turned out in this context that the effects of interaction stimulants like personalized and non-personalized tips, pointers to information sources, and mechanisms used in online treatments for tinnitus (e.g., in iCBT) should be further investigated.

## Introduction

The interaction of users with mHealth apps has been the subject of several investigations. Some mHealth apps simply monitor user activity, while others rely on active forms of interaction. In this work, we concentrate on active engagement with a mHealth app, for the recording of longitudinal *Ecological Momentary Assessments* (EMA)^1^.

EMA is an instrument used in psychology, social sciences and medicine, intended to collect momentary snapshots of behaviour or performance of the individuals under investigation, in real life conditions. The term “ecological” stresses the high ecological validity of such data^1^. The older term “experience sampling”, used by Csikszentmihalyi and Larson as early as 1978, reflects the use of the method for the investigation of highly fluctuating motivational states^2^, while the term “ambulatory asssessment”^3^ stresses the fact that data collection can be done in real time and in real life. May et al.^4^ performed a systematic literature review for EMA methodology in people with chronic pain. They pointed out that the study of chronic pain depends mainly on self-reported pain intensity assessments, which fits to the principles of EMA. They identified 62 quantitative EMA research projects, with a total of 105 scientific publications. They also found that there is a trend towards using smartphones as data collection devices.

Among the studies which investigated EMA in mobile apps, Marcano et al. compared “self-administered survey questionnaire responses” via a mobile app to other forms of data collection^5^. Other studies compared EMA collected via a mobile app with the retrospective statements of the patients^6–9^. The Youth EMA System (YEMAS) allowed for the collection of “automated texted reports of daily activities, behaviors, and attitudes among adolescents”, making use of the popularity of texting technology in that age stratum^10^. A remarkable finding on the use of EMA in smartphones was reported by Probst et al.^8^: they compared the sociodemographics of patients of an outpatient tinnitus clinic to the sociodemographics of the users of an EMA-based app, and found that this app reached different strata with respect to age and time since tinnitus onset^8^.

For the success of EMA-based monitoring of patients’ condition, it is essential that patients comply with the guidelines on how and how often they should fill in the EMA questionnaires^11–13^. For example, Stone and Shiffman stressed that “success of an EMA study depends on a high degree of participant compliance with the sampling scheme protocol; the validity of the assessment scheme is threatened by noncompliance”^11^, while Shiffman et al. pointed out that “Missing assessments have the potential to bias the obtained sample of behavior and experience, especially if the missing data are nonrandom”^12^. In the same context, Jones et al. identified a limitation in EMA, namely “while there are considerable benefits to Ecological Momentary Assessment (EMA), poor compliance with assessment protocols has been identified as a limitation, particularly in substance users”^13^. Wen et al.^14^ presented a systematic review on compliance for EMA used with mobile technologies. The more recent systematic review of May et al. on EMA^4^ does not focus on compliance but it discusses completion rates for the reported projects.

Recent investigations on the role of mobile technologies in healthcare also study how these technologies contribute to an increase of medical adherence. Badawy et al.^15^ performed a systematic review of more than 1000 publications on how interventions based on text-messaging and smartphones can contribute to adherence with respect to medication for children and adolescents. They performed a following systematic review on the potential of those technologies for “adherence to preventive behavior in adolescents”^16^; this systematic review also covered randomized clinical trials. Text messaging for reminders, alerts and motivation, but also for education and prevention, is discussed in the systematic review of systematic reviews by Marcolino et al.^17^ who covered 371 review studies.

While features such as reminders, pointers to educational content and EMA-questionnaires can be easily implemented as mobile apps, patients’ adherence to use them is not guaranteed and becomes a task by itself. Scherer et al. analyzed engagement with mHealth apps^18^ and found a relationship between patient engagement and dropout likelihood. In their investigation^14^ on compliance to mobile EMA protocols for children and adolescents (age $\le 18$ years old), Wen et al.^14^ compared studies with clinical vs. non-clinical designs with respect to average compliance rates and found no significant difference (76.9% vs. 79.3%, $\text {P}=.29$). Previous studies focused explicitly on adolescents, e.g., Garcia et al.^10^, Badawy and Kuhns^15^ and Badawy and Kuhns^16^, and Dou et al.^19^ found that neither age nor sex had a significant impact on acceptance of smartphone health technologies for chronic disease management.

In our work, we use the term “adherence” to describe the extent to which users completed EMAs with smartphone-based mHealth services. We use the EMA service of the mHealth app TrackYourTinnitus (TYT)^7^ as an example, and we study the potential of machine learning methods in characterizing users who are more/less likely to stop interacting with the app. It must be stressed that TYT delivers *observational data*, i.e. the users have neither been recruited nor instructed to use the service in a particular way, and they were free to change the EMA prompt settings.

Tinnitus is a complex chronic disorder that has no uniform way of manifestation and generation^20^. It describes the conscious perception of an auditory sensation in the absence of a corresponding external stimulus^21^. Moreover, Cima et al. stated that the patients’ reaction is an important component of the disorder^22^. The potential of smartphone technology for tinnitus management has been discussed previously, e.g. by Henry et al.^23^, and several studies on smartphone-based EMA for tinnitus demonstrated that valuable insights can be obtained on how people experience their tinnitus in everyday life^6–8,24–28^.

The aforementioned studies mostly concentrated on app users, who have delivered many EMA recordings. We rather analyze the data of users who delivered few, as well as many, EMA recordings, in agreement with the finding that the absence of recordings may be informative^18^. Using this observational data, we investigate following research questions:

$$\mathbf{RQ }_\mathbf{adherence }$$:
How to quantify “adherence” with respect to EMA recorded with a smartphone-based service on the basis of *observational* data, thereby taking into account that some users who interrupt interaction with the app may return later?
**RQ1**: How to frame the behavioral patterns of mHealth app users with respect to their adherence with the app?
**RQ2**: To what extent can we predict discontinuation of app usage based on the first days of interaction?
**RQ3**: To what extent can we predict user adherence from their data at registration, i.e., before they start interacting with the app?

Authors typically quantify adherence as the proportion of non-self-initiated prompts that received a participant response^4,14^; May et al. refer to this proportion as “response” rate^4^, Wen et al. as “compliance” rate^14^. We rather use two measures; duration and continuity, which we introduce formally as indicators of *adherence*. Informally, under “duration” we count how long a user keeps answering the non-self-initiated prompts over a specified time period. Under “continuity” we capture periods of unanswered prompts during this time period. For $\text {RQ}_{{\mathrm{adherence}}}$, we quantified adherence across these two dimensions. For RQ1 and RQ2, we analyzed the multi-dimensional time series of the users’ EMA recordings. For RQ3, we analyzed the answers to the questionnaires they filled in at registration.

## Materials

A multidisciplinary European guideline for tinnitus^22^ from 2019 defined tinnitus as a perception of a sound or sounds without external sources, appearing in the ear or head. The authors pointed out that this phantom perception becomes a problem for some of the affected persons and named this form “bothersome (distressing) tinnitus”^22^. Cima^29^ described this type as a negative emotional and auditory experience, associated with, or described in terms of, actual or potential physical or psychological harm^22,29^. Furthermore, patient profiles are described as very heterogeneous due to the highly complex nature of tinnitus with its multi-factorial origins^20,22^. Treatments with a curative effect are not available in most cases^21,22,30^. Therefore, some authors such as Tyler et al.^31^, emphasize the necessity to identify subgroups and to investigate effective treatments for them.

Tinnitus has several aspects for which valuable data can be gathered through the use of mobile technology and EMA. Firstly, tinnitus patients are very heterogeneous, which makes it difficult to find general treatments. Secondly, patients usually pose challenging moment-to-moment variations, which are difficult to capture with traditional clinical trials and methods. Therefore, the TrackYourTinnitus platform was developed by an interdisciplinary team. Clinically, it was developed to gather longitudinal data based on an observational study design. Through the use of a sophisticated collection procedure^32^, TYT is able to provide a data source with many investigation opportunities. Most importantly for the work at hand, the condition of a user is captured when registering to the TYT app through the use of three mandatory questionnaires, that the new users have to answer sequentially during the process of registration on the TYT platform in order to create a user account (i.e., the baseline characteristics). Two of them are utilized in this work, namely the *Tinnitus Sample Case History Questionnaire* (TSCHQ, German version)^33^, as well as the *Mini Tinnitus Questionnaire* (MiniTF)^34^. As the MiniTF cannot be found in an English version in the literature, it is provided in the appendix^24^ (“Supplementary A—Table 1”).

The study was approved by the Ethics Committee of the University Clinic of Regensburg (ethical approval #: 15-101-0204). All users read and approved the informed consent before participating in the study. The study was carried out in accordance with relevant guidelines and regulations.

### EMA during interaction with the mHealth app

Table 1 shows the items of the EMA questionnaire of TYT^26^, which the users must fill in more than once a day. Dichotomous questions were answered with Y (yes) or N (no). The other questions were answered using a Visual Analog Scale (VAS)^26^. For our analysis, we skipped the 8th item due to an ambiguity in the recordings, hence we have a 7-dimensional time series per user.

**Table 1:** EMA questionnaire of TYT^26^.

Item	Translation of the German question
q1	Did you perceive the tinnitus right now? (Y/N)
q2	How loud is the tinnitus right now?
q3	How stressful is the tinnitus right now?
q4	How is your mood right now?
q5	How is your arousal right now?
q6	Do you feel stressed right now?
q7	How much did you concentrate on the things you are doing right now?
q8	Do you feel irritable right now? (Y/N)

### Collection of the observational data

The complete database of the TYT app recordings contains EMAs from April 10 2014 onwards, supplemented by the questionnaire answers filled in by the users at registration. For our study, we acquired a data export for the period April 10 2014 to February 3 2017. These recordings stem from 1292 users (338f/908m/46u). The countries with the most registered users were Germany (457), US (188), GB (75), and The Netherlands (75). The average age at tinnitus onset was 35.51 years (SD: 14), while the average age at the moment of the registration was 44.08 years (SD: 13.25). The average number of years between tinnitus onset and registration was 9.02 years (SD: 10.98). This dataset, denoted as D[1292] hereafter, was used as the basis for our analysis. For the individual research questions, we applied further exclusion criteria as follows:

- For RQ1, we used the complete D[1292].
- For RQ2, we removed users that had only one EMA recording. 440 users were thus removed, retaining 852 users (230f/592m/30u) with an average age of 35.27 years (SD: 14.31) at tinnitus onset, an average age of 44.71 years (SD: 13.07) at the moment of registration and 9.37 years on average between tinnitus onset and registration (SD: 11.36). We denote this dataset as D[1292:852].
- For RQ3, we used both the D[1292] and a subset of the D[1292:852], produced after fixing the horizon of observations to N = 30 days. Ten users were excluded from D[1292:852], because their time series started at the end of the export. We denote this dataset as D[1292:842] with 842 users (227f/585m/30u) with an average age of 34.89 years (SD: 15.24) at tinnitus onset, an average age of 43.95 years (SD: 14.08) at the moment of registration and 8.90 years on average between tinnitus onset and registration (SD: 11.32). Furthermore, 26 users were removed, because they have missing values for age or age at tinnitus onset. Therefore, 816 users remained (219f/567m/30u) with an average age of 35.83 years (SD: 14.31) at tinnitus onset, an average age of 44.81 years (SD: 13.11) at the moment of registration and 9.04 years on average between tinnitus onset and registration (SD: 10.96). We denote this dataset as D[1292:816].

## Methods

To quantify the user interaction with the mHealth app, we defined a two-dimensional concept of “adherence”, and we used these dimensions as target variables for classification (RQ2 and RQ3). To address RQ1, we expressed the interaction of a user as a set of sequences of EMA recordings, one sequence per item of the EMA questionnaire (cf. Table 1). Hence, we turned the activities of the users into time series. Accordingly, for RQ2, we used time series classification. For RQ3, which concerns the static data at registration (MiniTF and TSCHQ), we used classification rules induction. We evaluated the derived models on classification accuracy.

### A multi-dimensional definition of adherence

The mHealth app TYT allows the user to specify how many times per day s/he wants to fill in the EMA questionnaire. Each time, the user may fill out the whole questionnaire or skip some items. We specify that on a given day a user has performed an “EMA entry” only if they responded at least once to the TYT notification and has filled in at least one questionnaire item. On this basis, we propose the following definitions:

- *Interaction duration:* number of days in which a user has performed EMA entries during the observation period
- *Interaction continuity* encompasses
  - *Days until first break (FirstDays):* number of adjacent days with EMA entries from the day of registration onwards, until the first “break” in the user’s interaction was encountered, i.e., the first day without EMA entry.
  - *Return after first break (Return):* dichotomous variable (Yes/No), determining whether the user returns, i.e., resumes the use of the app after the first break (Yes) or not (No).

Interaction duration thus captures the number of days of user–app interaction (maximally: the whole observation period), allowing for breaks during which the user has no EMA entries.

Interaction continuity captures breaks: if a user does not return after the first encountered break, then the interaction duration is equal to the number of adjacent days until break (and can be as low as 1).

It is noted that the values of interaction duration and interaction continuity are determined inside an *observation period*. Since TYT users may start interaction on any calendar day, we aligned all users’ first EMA entry as Day 1. Since some users had very long breaks in interaction, we collapsed all days with EMA entries into a sequence of non-adjacent days and used the *FirstDays* value to determine whether this sequence consists of adjacent days or not. For our study, we specified an observation period (H) of 30 days.

### Modeling adherence with a user’s temporal and static data

For the $j$th EMA item $j=1\ldots {}J_{EMA}$, we denote as $TS_{u,j}[t]$ the set of EMA recordings for this item at the $t$th day of interaction of user *u* with the app. All users are aligned at $t=1$, so that $TS_{u,j}[1]$ denotes the answers to the $j$th EMA item on the 1st day of interaction. The observation period encompasses *N* days.

We denote as $TS_{u,j}$ the time series of *u* for the $j$th EMA item and as $n_{u,j}$ the length of this time series. If $n_{u,j}<N$, then $TS_{u,j}[t]$ is NULL for $t>n_{u,j}$. Since *u* may selectively fill values for the different EMA items, the value of $n_{u,j}$ varies with *j*, we specify that the interaction duration of *u* within the observation horizon is $Duration _u=\max _{j=1\ldots {}J_{EMA}}n_{u,j}$.

We further denote as ${\textit{FirstDays}} _{u,j}$ the number of days until first break for the $j$th EMA item. This means that the entries in $TS_{u,j}[t]$ for $t\le {\textit{FirstDays}} _{u,j}$ are adjacent. For user *u*, we specify that ${\textit{FirstDays}} _u=\max _{j=1\ldots {}J_{EMA}} {\textit{FirstDays}} _{u,j}$. Accordingly, the dichotomous variable ${\textit{Return}}_u$ is set to Yes, if $Duration _u> {\textit{FirstDays}} _u$ and to No otherwise.

Next to the temporal data that express the interaction of *u* with the app, we also consider the questionnaires filled in during registration. Hence, *u* is modeled as vector with four elements as follows:

$$\begin{aligned} MiniTF _u, \quad TSCHQ _u, \quad \{(n_{u,j}, {\textit{FirstDays}} _{u,j},TS_{u,j})|j=1\ldots {}J_{EMA}\}, \quad {\textit{Return}}_u \end{aligned}$$

Since the number of recordings per EMA item at a given day may vary across users, we collapse the set $TS_{u,j}[t]$ for $t\le {}n_{u,j}$ into the mean of the values observed for that day.

For RQ2 and RQ3, we use *Return* as the target variable and train classifiers that separate between users who return after the first break from those that do not return. For RQ2, we consider the time series data. For RQ3, we consider the responses to the MiniTF and the TSCHQ items.

### RQ2 as a time series classification task

We formulate RQ2 as the following time series classification task: among users with the same value of *FirstDays*, how well can we separate between those that stopped and did not come back (${\textit{Return}}=\text {No}$), and those that did come back again later (${\textit{Return}}=\text {Yes}$)?

#### RQ2 as classification task on strata of time series

We used a time period of *N* days and a set of time series ${\mathscr {T}}$, where the time series of a user *u* has length $Duration _u\le {}N$; entries after the $N$th are ignored. We set a cut-off $\tau _{early}\ll {}N$ for the first, early days of interaction and stratified the time series on length. For each length stratum $k=1\ldots \tau _{early}$, we built the sets of users $U_{k,{\textit{Return}}=No}$ and $U_{k,{\textit{Return}}=Yes}$:

- User *u* belongs to $U_{k,{\textit{Return}}=No}$ if and only if: $Duration _u= {\textit{FirstDays}} _u=k$.
- User *u* belongs to $U_{k,{\textit{Return}}=Yes}$ if and only if: ${\textit{FirstDays}} _u=k$ and $Duration _u> {\textit{FirstDays}} _u$.

For each stratum (value $k=1\ldots \tau _{early}$), we trained classifiers that separate between users who stopped the interaction after *k* adjacent days of interaction (${\textit{Return}}=\text {No}$), and those that returned after the first break (${\textit{Return}}=\text {Yes}$). To identify properties that characterize users who returned after the first break, we investigated the discriminative power of the individual EMA items by training a separate classifier for the time series of each EMA item. Training separate classifiers also allowed us to take into account that some users did not fill all EMA items, i.e., some EMA answers may be Missing Not At Random. Training on strata also allowed us to adjust our evaluation measure (described later) to take into account that the proportions of ${\textit{Return}}=\text {Yes}$ vs. ${\textit{Return}}=\text {No}$ change with stratum length.

#### Algorithms for classification of time series strata

We took as a basis the comprehensive evaluation of algorithms by Bagnall et al.^35^ and chose the following well-performing algorithms: Shapelet Transform (ST)^36^, Time Series Forest (TSF)^37^, Elastic Ensemble (EE)^38^, Move–Split–Merge (MSM)^39^, Complexity Invariant Distance (CID)^40^, Derivative Transform Distance ($\text {DTD}_{{\mathrm{C}}}$)^41^, and Derivative DTW ($\text {DD}_{{\mathrm{DTW}}}$)^42^. We used the Java implementation offered by Bagnall et al.^35,43^, with the original parameter settings of the algorithms (see also^43^).

For the use of these algorithms, the time series must be univariate, aligned, of the the same length, and without missing values^35^. All these assumptions were satisfied in our study. In particular, for each user *u*, the time series $TS_{u,j}, j=1\ldots {}J_{EMA}$ were aligned as $TS_{u,j}[t]$ referring to the first EMA entry of the user, independently of the exact time point; they have no missing values because the $t$th day of interaction and the $t+1$st day of interaction need not be adjacent; although the complete time series of the users are not of the same length, the inputs to a classifier are, because we train classifiers separately for each stratum—here for each value of *FirstDays*.

Next to the aforementioned dedicated algorithms for time series, we also studied the performance of classification algorithms that are not dedicated to time series, namely: Rotation Forest (RotF)^44^, Random Forrest (RandF)^45^, C45^46^, Naïve Bayes (NB)^47^, 1-Nearest-Neighbors (1NN) based on a distance measure of Euclidean distance (ED), and 1NN using dynamic time warping (DTW)^48^). For these algorithms, the *k* observed EMA values per EMA item in the stratum of length *k* were treated as values of independent observations, ignoring their order.

#### Classifier evaluation with changing class priors

For each $k=1\ldots {}m$, we partition the set $U_{k,{\textit{Return}}=Yes}\cup {}U_{k,{\textit{Return}}=No}$ into a training set and a test set using the “stratified holdout” method of the R library *rminer*^49^ with a 2:1 ratio (i.e., two thirds of the labeled data are used for training and one third for testing) and with seed = 145. As the basic evaluation criterion, we used accuracy. Since the priors of the two classes may change with the value of *k* (in D[1292:852], the majority class is even reversed from $k=1$ to $k=2$), we set as baseline for accuracy the prior of the majority class and discarded classifiers that achieved an improvement of less than $\tau _{improve}$ percentage units.

### RQ3 as classification task

We map RQ3 into a classification task for ${\textit{Return}}=\text {Yes/No}$. Since ‘${\textit{Return}}=\text {Yes}$” implies an interruption in the interaction of the user with the mHealth app, we focus on identifying items in the registration questionnaire, which predict discontinuous interaction (i.e., interruption and return) with high discriminatory power.

For classification rule induction, we skipped users who had missing values for some of the questionnaire items: 826 users were retained (452 with ${\textit{Return}}=\text {Yes}$, 364 with ${\textit{Return}}=\text {No}$).

Classification rule induction is based on the a priori algorithm for association rule discovery, first introduced in 1993^50^: these rules have the form “IF B THEN H”, or equivalently $B\rightarrow {}H$, where the rule antecedent B consists of features, i.e. (item, value range) pairs, connected by a logical operator like “AND” (denoted as “&” or “$\wedge$”), while the rule consequent H contains the class to be predicted^51^. Since we were mainly interested in interruption and return, we induce rules for *Return* = Yes, whereupon the features are the answers to the items of the MiniTF and TSCHQ.

Conventional classification rules discovery algorithms are restricted to non-numerical data only. Rules involving numerical features are supported by the HotSpot rule discovery algorithm^52^. In our study, we use the Interactive Medical Miner software^53–55^, which supports both conventional classification rules and HotSpot rules.

Rule discovery algorithms are typically evaluated on $support (\cdot )$, $precision (\cdot )$ and $lift (\cdot )$, which are defined as follows^56^:

- $support (B\rightarrow {}H=P(\{B,H\})$, i.e., the likelihood of observing the antecedent *B* and the consequent *H* together
- $$precision (B\rightarrow {}H)=\frac{ support (B\rightarrow {}H)}{ support (B)}$$
- $lift (B\rightarrow {}H)=\frac{P(H|B)}{P(H)}$, where lift values of less than 1 indicate that the consequent counteracts with the antecedent, the value of 1 indicates that the consequent is independent of the antecedent; hence, we concentrate on values larger than 1.

For our study, we set the support lower boundary to 1% and the maximum rule length to 2. We further constrained the rule induction process so that the maximum number of child rules derived from a rule is 800, the minimum gain in precision is 0.01. We retained only rules with a p-value of less than 0.05 (without correction for multiple testing). These thresholds already led to a substantial reduction of the set of induced rules that are subjected to further inspection.

### Software for individual analysis tasks

Following packages of the statistical software “R”^57^ were used for most of the analysis: rpart^58^, rattle^59^ and rminer^49^.

## Results

We used D[1292] to identify patterns of interaction (RQ1), to predict adherence on the basis of the first days of interaction (RQ2), and to explain the discontinuation of interaction on the basis of static data (RQ3).

### RQ1 on D[1292]: patterns of interaction duration

**Figure 1:**
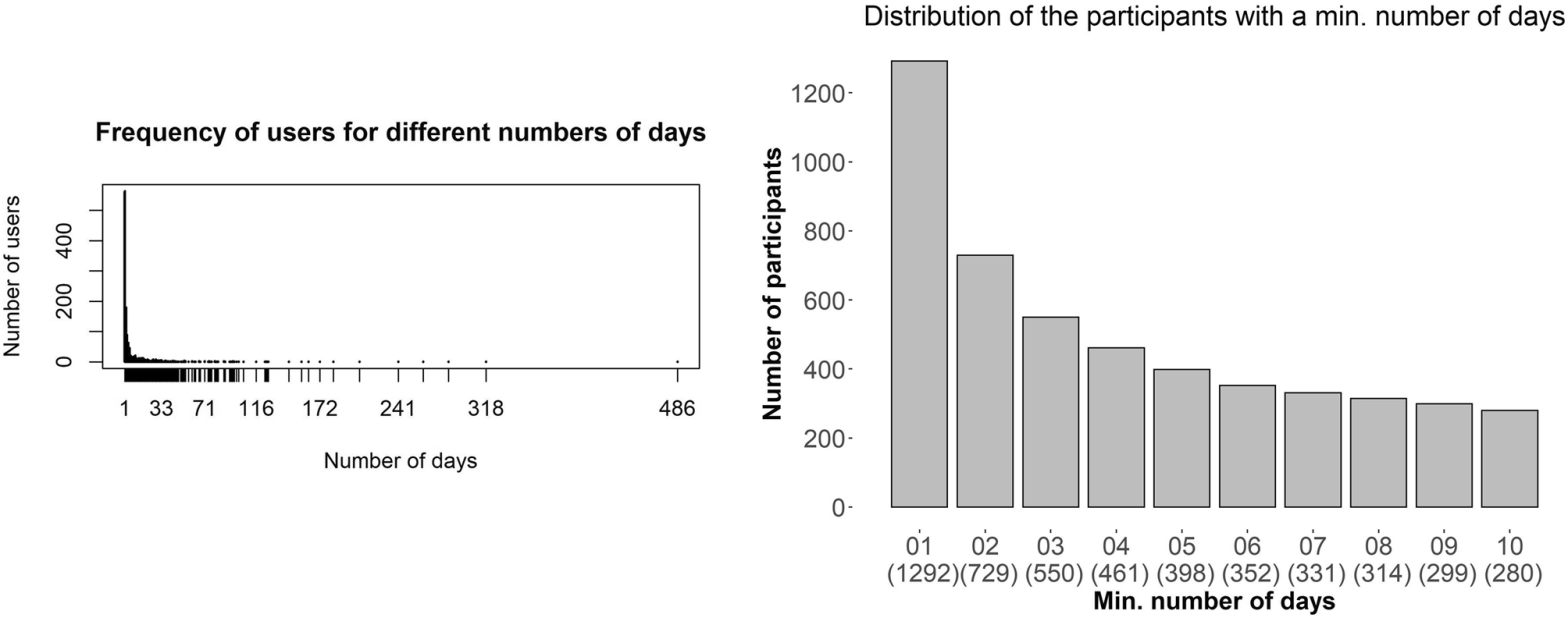
Two histograms of interaction duration on D[1292:852]: The number of users who recorded a given number of EMA entries decreases rapidly as this number increases above 1 (left-hand part); the number of users who recorded a given number of EMA entries as minimum decreases more slowly from 1 towards 10 (right-hand part).

Figure 1 shows high-level patterns of interaction with the mHealth app in the form of histograms. The left-hand part of the figure depicts the distribution of *interaction duration* among the TYT users. The right-hand part of the figure condenses the previous histogram by counting the number of users who have at least one, two, ..., and $\tau _{early}+1$ EMA entries. It is stressed that since we defined an “EMA entry” as a day in which an EMA recording was made, counting days is equivalent to counting EMA entries.

As expected, the number of users who entered EMA data more than once decreased quickly, but there were some users with a substantial number of EMA entries (see right area of the plot in the left-hand part of Fig. 1). 852 users (230f/592m/30u) had more than one day with EMA entries. This is sufficient for all time series classification algorithms to learn on, except one (ST). ST needs at least three days of data as we defined the time series. This means that there must be at least two EMA entries in total, one of which is not on the first day and one more entry at a third day.

The decrease in the number of users slows down when moving from a minimum of two EMA entries to a minimum of three EMA entries: more than 42% of the users had an *interaction duration* of three or more days, independently of the *interaction continuity*.On the other hand, 280 users entered EMA data at least 10 times (see right area of Fig. 1).

### RQ2 on D[1292:852]: predicting interaction continuity from the early interaction data

For each user *u*, we firstly computed the value of ${\textit{FirstDays}}_u$. Then, setting the horizon of observation to $N=30$ days, we checked for each *u* with a *FirstDays* of less than *N* (i.e., for each user who had interrupted the interaction) whether this user returned again within the *N* days (${\textit{Return}}_u=\text {Yes}$) or not (${\textit{Return}}_u=\text {No}$). For “early interaction”, we set the cut-off $\tau _{early}$ equal to 9 days. This means that we made no distinction among users with *FirstDays* $=\tau _{early}+1,\tau _{early}+2,\ldots ,N$.

#### Interplay between the value of *FirstDays* and the likelihood of *Return*

The interplay between the value of *FirstDays* and the value of the dichotomous variable *Return* is depicted in Table 2.

**Table 2:** For each value of *FirstDays*, number of participants who did not vs. did return within the horizon of $N=30$ days, sorted by EMA item; the likelihood of the majority class for each value of *FirstDays* and EMA item is shown in parentheses.

${\textit{FirstDays}}=$	1	2	3	4	5	6	7	8	9	$\ge {}10$
EMA item#	Number of participants with $\text {Return}=\text {No}$ (left) vs. $\text {Return}=\text {Yes}$ (right)									
q1	22 vs. 158	87 vs. 97	58 vs. 35	48 vs. 22	40 vs. 12	34 vs. 3	32 vs. 2	34 vs. 3	52 vs. 4	58 vs. 41
	(0.88)	(0.53)	(0.62)	(0.68)	(0.77)	(0.92)	(0.94)	(0.92)	(0.93)	(0.58)
q2	26 vs. 155	82 vs. 97	57 vs. 35	51 vs. 22	37 vs. 12	33 vs. 3	33 vs. 2	33 vs. 3	53 vs. 3	58 vs. 41
	(0.85)	(0.54)	(0.62)	(0.7)	(0.75)	(0.92)	(0.94)	(0.92)	(0.93)	(0.58)
q3	28 vs. 154	84 vs. 97	56 vs. 34	47 vs. 22	39 vs. 12	34 vs. 3	32 vs. 2	34 vs. 4	52 vs. 3	58 vs. 40
	(0.85)	(0.54)	(0.62)	(0.68)	(0.76)	(0.92)	(0.94)	(0.89)	(0.95)	(0.59)
q4	24 vs. 153	86 vs. 99	57 vs. 36	46 vs. 22	42 vs. 12	33 vs. 3	32 vs. 2	33 vs. 3	56 vs. 3	54 vs. 41
	(0.86)	(0.53)	(0.61)	(0.68)	(0.78)	(0.92)	(0.94)	(0.92)	(0.95)	(0.57)
q5	26 vs. 153	90 vs. 96	52 vs. 36	47 vs. 22	38 vs. 12	36 vs. 2	32 vs. 2	33 vs. 3	53 vs. 3	56 vs. 41
	(0.85)	(0.52)	(0.59)	(0.68)	(0.76)	(0.95)	(0.94)	(0.92)	(0.95)	(0.58)
q6	30 vs. 155	86 vs. 96	52 vs. 34	46 vs. 22	38 vs. 12	33 vs. 3	37 vs. 2	33 vs. 3	52 vs. 4	55 vs. 40
	(0.88)	(0.53)	(0.6)	(0.68)	(0.76)	(0.92)	(0.95)	(0.92)	(0.93)	(0.58)
q7	26 vs. 155	87 vs. 100	57 vs. 35	46 vs. 21	37 vs. 11	34 vs. 4	35 vs. 1	33 vs. 3	52 vs. 3	56 vs. 40
	(0.85)	(0.53)	(0.62)	(0.69)	(0.77)	(0.89)	(0.97)	(0.92)	(0.95)	(0.58)

A cell in Table 2 contains the numbers of users with ${\textit{FirstDays}}=1,2,\ldots \tau$. In particular, the first value (for ${\textit{Return}}=\text {No}$) refers to the set of users that interacted without interruption and then gave up, whereupon their interaction duration is also equal to their *FirstDays* value. The second value refers to the users who interrupted after *FirstDays* but then returned to the app at a later time point (${\textit{Return}}=\text {Yes}$).

Since a user *u* may decide to answer only some of the EMA items, the value of ${\textit{FirstDays}}_{u,j}$ may vary with the EMA item $j=1\ldots {}K_{EMA}$. For example, the column for ${\textit{FirstDays}}=2$ (see 3rd column of Table 2) shows that 87 users answered q7 for the first and second day, and then stopped (for q7, see last row of the table), while only 86 users answered q6 for the first 2 days before stopping (see row previous to the last one). Similarly, after 2 days, 100 users interrupted but then returned to the app to answer q7 again; for q6, only 96 users returned. These differences are not substantial though. In contrast, there are large differences in the values across columns: the number of users that interrupted after *FirstDays* days drops quickly; from more than 150 for ${\textit{FirstDays}}=1$ to less than 5 for ${\textit{FirstDays}}=6$. The number of users who did not return after *FirstDays* days (${\textit{Return}}=\text {No}$) increases from ${\textit{FirstDays}}=1$ to ${\textit{FirstDays}}=2$, and then starts decreasing slowly. Hence, as the value of *FirstDays* increases, the likelihood that the user will quit altogether becomes higher than the likelihood of interrupting and then coming back again.

#### Influence of EMA items on classification quality

For an $N=30$ days long observation period, with a cut-off for “early interaction” $\tau _{early}$ set to 9 days, for each EMA item $j=1,\ldots ,K_{EMA}$, and for each of the 13 classification algorithms, we induced one model per ${\textit{FirstDays}}=1,2,\ldots ,\tau$ and one for ${\textit{FirstDays}}\ge \tau +1$. Since $K_{EMA}=7$, this resulted in $7\times {}10=70$ models for each of 11 out of the 13 algorithms. Algorithm EE (the elastic ensemble) requires time series with at least two time points, while algorithm ST requires at least three time points, so we built fewer models accordingly.

For accuracy improvement over the prior distribution of the majority class, we took the prior of the majority class for ${\textit{FirstDays}}=6$ as a basis, which is 92% for most of the EMA items: the maximal improvement is $100\%-92\%=8\%$ which led to $\tau _{improve}=8$ percentage units. The original accuracy values are part of the Supplementary Material, namely “Supplementary B” Table 2 (first 6 algorithms) and Table 3 (remaining 7 algorithms).

Table 3 depicts the *FirstDays* values and EMA items for which some models achieved a quality improvement. There was no improvement for the ${\textit{FirstDays}}=1,5,6,7,8,9$, i.e., for the strata with a very skewed distribution towards either of the two *Return* values. Despite the fact that no algorithm was tuned towards skew, the dedicated time series classification algorithms, as well as RotF, NB, and ED did achieve quality improvements of at least $\tau _{improve}$ percentage units.

**Table 3:** Models with an improvement of at least $\tau _{improve}$ percentage units over accuracy ($\text {Return}=\text {Yes/No}$) in dependence of *FirstDays* value (columns) and EMA item (row), for a horizon of observations of 30 days and a cut-off $\tau _{early}=9$ days.

Algorithm	EMA item	*FirstDays*			
		2	3	4	$\ge 10$
ST	q1	–	0.71	$\times$	$\times$
	q5	–	$\times$	0.83	$\times$
	q6	–	0.68	$\times$	$\times$
TSF	q1	0.61	$\times$	$\times$	$\times$
	q5	0.61	0.69	$\times$	$\times$
EE	q5	0.66	$\times$	$\times$	$\times$
MSM	q5	0.63	$\times$	$\times$	$\times$
$\text {CID}_{{\mathrm{DTW}}}$	q1	0.61	$\times$	$\times$	$\times$
	q5	0.63	$\times$	$\times$	$\times$
$\text {DTD}_{{\mathrm{C}}}$	q1	0.62	$\times$	0.78	$\times$
	q2	$\times$	$\times$	$\times$	0.67
	q5	0.61	$\times$	$\times$	$\times$
$\text {DD}_{\mathrm{DTW}}$	q1	0.61	$\times$	$\times$	$\times$
	q5	0.61	$\times$	$\times$	$\times$
DTW	q2	$\times$	$\times$	$\times$	0.67
	q5	0.63	$\times$	$\times$	$\times$
RotF	q1	$\times$	0.71	$\times$	$\times$
	q5	$\times$	0.69	$\times$	$\times$
NB	q4	$\times$	$\times$	$\times$	0.66
	q5	$\times$	0.69	$\times$	$\times$
	q7	$\times$	0.71	$\times$	$\times$
ED	q1	0.62	$\times$	0.78	$\times$
	q2	$\times$	$\times$	$\times$	0.67
	q5	0.63	$\times$	$\times$	$\times$

Table 3 shows that some EMA items contribute well to class separation. EMA item “q5” exhibits the most important contribution, since 11 out 13 models on this item can separate well between ${\textit{Return}}=\text {Yes}$ and ${\textit{Return}}=\text {No}$ at very early stages, namely for an interruption after ${\textit{FirstDays}}=2,3$ or 4 days. EMA item “q1” also contributes greatly to separation, since 7 of the models on this item can separate between returning and non-returning users, again for ${\textit{FirstDays}}=2$, 3, 4. Models on EMA item “q2” contribute to separation for ${\textit{FirstDays}}\ge 10$. There are models on EMA items “q4”, “q6” and “q7” that lead to satisfactory separation, but only for few *FirstDays* values, while models on EMA item “q3” did not contribute to good model separation.

Hence, we conclude that the responses on the EMA items “q1” (tinnitus perception) and “q5” (arousal) in the first days of interaction can give indication of whether the user will discontinue interaction or return after some time.

### RQ3 on D[1292:842]: user attitude at registration (MiniTF) predicts interaction continuity

We have induced classification rules on MiniTF to identify the static properties characteristics of the users who returned after interrupting their use of the mHealth app (*Return* = Yes). Ten of the users in D[1292:852] were excluded because their time series started at the end of the export, as explained under “Materials”.

The induced classification rules for ${\textit{Return}}=\text {Yes}$ on MiniTF are depicted in Table 4. They are sorted based on the descending value of lift. By definition of lift, a subpopulation (rule antecedent) with a lift of more than 1.0 is more likely to return after interruption (${\textit{Return}}=\text {Yes}$) than is the case for the whole population under study. If, additionally, the precision is equal to 1.0, then the consequent (${\textit{Return}}=\text {Yes}$) holds for all users described by the antecedent, i.e., all users of this subpopulation will return to using the app after they interrupted. This is the case for the first rule in Table 4.

**Table 4:** Classification rules for ${\textit{Return}}=\text {Yes}$ on MiniTF with the following parameter settings: support lower boundary $= 0.01$, maximum rule length $= 2$, maximum number of child rules $= 800$, minimum gain in precision $= 0.01$, p-value $< 0.05$ (without correction for multiple testing).

Classification rules		Support	Precision	Lift
$${\varvec{Return}}=\varvec{Yes}$$				
(1)	$\text {tf5} = 2$ & $\text {tf10} = 0$	0.01	1	1.81
(2)	$\text {tf5} = 2$ & $\text {tf1} = 1$	0.06	0.74	1.34
(3)	$\text {tf8} = 2$ & $\text {tf10} = 1$	0.05	0.73	1.32
(4)	$\text {tf8} = 0$ & $\text {tf10} = 0$	0.05	0.71	1.29
(5)	$\text {tf8} = 0$ & $\text {tf11} = 0$	0.14	0.66	1.2
(6)	$\text {tf5} = 2$ & $\text {tf2} = 1$	0.11	0.66	1.2
(7)	$\text {tf7} = 2$ & $\text {tf6} = 1$	0.15	0.64	1.16

The categorical items are encoded with $2 = \text {True}$, $1 =$ Partially True, and $0 =$ False.

By the nature of MiniTF, a value of 2 (True) for some questions indicates a worry of the users. Three of the rules depicted on Table 4, namely the 1st, 2nd, and 6th, refer to users with worries on the effects of tinnitus on their physical health ($\text {tf5}=2$). The 3rd rule refers to users who have difficulties in sleeping ($\text {tf8}=2$), while the 7th one refers to users whose tinnitus signal is so disturbing that they cannot ignore it ($\text {tf7}=1$). In contrast, the 4th and the 5th rules refer to users that do not have difficulties in sleeping ($\text {tf8}=0$), and have a more positive attitude towards the disease ($\text {tf10}=0$ or $\text {tf11}=0$ or both).

These rules indicate that users who return after the first interruption do not constitute a homogeneous subpopulation, but vary substantially in how they experience their tinnitus. Nonetheless, concerns about physical health, difficulties with sleep, and disturbance through tinnitus loudness, as captured by MiniTF, are predictive of the continuation of the interaction after the first interruption.

### RQ3 on D[1292:842]: interaction continuity does not depend on MiniTF scores

Only 697 of the users in D[1292:842] interacted with the mHealth app for more than one day. From the 842 users, 465 returned after the first interruption (${\textit{Return}}=\text {Yes}$), and 377 did not (${\textit{Return}}=\text {No}$). The juxtaposition of the scores for the two user groups (cf. Table 5) indicates that there is no difference in the likelihood of observing a specific score range within each group.

**Table 5:** MiniTF scores at registration for 842 users who returned after the first interruption and those that do not.

*MiniTF score*	*n*	${\textit{Return}}=\text {Yes}$	${\textit{Return}}=\text {No}$
0–7	103	56	47
8–12	170	93	77
13–18	284	164	120
19–24	221	111	110
NA	63	41	23

### RQ3 on D[1292:816]: some user characteristics captured in TSCHQ are associated with interaction continuity and duration

We induced classification rules for ${\textit{Return}}=\text {Yes}$ onto the answers to the TSCHQ questionnaire, which is more detailed than MiniTF and also captures sociodemographics and physiological aspects of tinnitus, next to tinnitus perception. To assess the association of the TSCHQ answers with the duration of interaction, we also used the NumDays variable, which counts the *total* days of interaction with TYT, independently on whether or not they were within a 30-days horizon of observation. As described in “Materials”, 26 more users were excluded from D[1292:842] because of missing values for the variables age or age at tinnitus onset.

Table 6 shows the induced classification rules, organized into two rule sets: the rule set in the upper part of the Table encompasses rules characterized by the users’ answers at registration, while the rule set in the lower part of the Table encompasses rules that include the NumDays from the interaction data. Within each group, the rules are sorted on lift descending, then on precision and then on support. It is noted that due to the computation algorithm for HotSpot rules and due to the constraint on rule length, some of the induced rules may overlap: Rules 1) and 2) refer to the same users, and rules 3) and 4) are likely to refer to the same persons, too.

**Table 6:** Classification rules for ${\textit{Return}}=\text {Yes}$ over TSCHQ and NumDays with the following parameter settings: support lower boundary $= 0.01$, maximum rule length $= 2$, maximum number of child rules $= 800$, minimum gain in precision $= 0.01$, p-value $<0.05$ (without correction for multiple testing).

Classification rules		Support	Precision	Lift
(1)	ageAtRegistration $> 68$ & sex $= 1$	0.01	1	1.81
(2)	ageAtRegistration $> 67$ & sex $= 1$	0.01	1	1.81
(3)	ageAtRegistration $> 67$ & onsetrelation $= 3$	0.01	1	1.81
(4)	ageAtRegistration $> 65$ & familyHistory $= 1$	0.01	1	1.81
(5)	ageAtRegistration $> 68$ & variability $= 1$	0.02	0.92	1.66
(6)	ageAtRegistration $> 68$ & ageAtOnset $\le 68$	0.01	0.92	1.66
(7)	NumDays $> 9$ & ageAtRegistration $\le 27$	0.02	1	1.81
(8)	NumDays $> 3$ & onsetrelation $= 4$	0.02	1	1.81
(9)	NumDays $> 9$ & ageAtOnset $> 58$	0.01	1	1.81
(10)	NumDays $> 9$ & onsetrelation $= 4$	0.01	1	1.81
(11)	NumDays $> 9$ & NumDays $\le 10$	0.03	0.95	1.72
(12)	NumDays $> 2$ & ageAtRegistration $> 68$	0.02	0.93	1.68
(13)	NumDays $> 5$ & familyHistory $= 1$	0.1	0.91	1.64
(14)	NumDays $> 9$ & familyHistory $= 1$	0.07	0.9	1.62
(15)	NumDays $> 20$ & onsetrelation $= 3$	0.05	0.88	1.59

The users in the rule set in the upper part of the Table 6 constitute small groups of elderly users (ageAtRegistration $> 65$ for all antecedents). The first four rules refer to elderly female users whose tinnitus was caused by a trauma (rule 3) and without further incidents of tinnitus in the family history (rule 4). Rule 5) refers to users whose tinnitus does not vary during the day, while rule 6) refers to users that have had tinnitus for more than a year. These elderly users are very likely to return after an interruption in their interaction—as indicated by rule 12).

The antecedents in the rule set in the lower part of the Table 6 refer to NumDays of interaction. The rules indicate that the users who return after an interruption constitute small and very different groups: among those who interact for more than 9 days are young users (of 27 years or less, see rule 7), and users of at least 59 years of age (since they were 58 when the tinnitus started, see rule 9). Some users had tinnitus after a change in hearing ($\text {onsetrelation}=4$, see rules 8 and 10) and others after a head trauma ($\text {onsetrelation}=3$, see rule 15).

The two rule sets in combination indicate that there is a homogeneous group of elderly users whose tinnitus has been caused by trauma and who interact with the app for more than 2 days and perhaps much longer (see rule 15). The younger users constitute less homogeneous groups.

## Discussion

There are a large number of investigations on smartphone-based EMA for psychosomatic disorders. Linardon et al. point out that “although the efficacy of smartphone-delivered interventions for mental health problems is emerging, randomized controlled trials (RCTs) of smartphone interventions are characterized by high rates of attrition and low adherence”, and they report a “mean meta-analytic study attrition rate [of] 24.1% (95% CI [19.3, 29.6]) at short-term follow up”^60^. Our findings on the duration of interaction among the participants in D[1292] are even more severe, since only 852 of the TYT users (ca. 68%) had more than one EMA. This is close to the feasibility study of Henry et al. on tinnitus management^23^, where it was observed that the app usage drastically dropped after the first day of interaction.

Since our analysis is on observational real life data rather than on data from participants recruited for a study, the actual values are not comparable, but the insight remains: a non-negligible subset of users gives up very early on, while those who continue recording their EMA do so with deteriorating intensity.

In an analysis on adherence to EMAs performed by Colombo et al.^61^ for depression, it was found that eleven out of thirteen analyzed studies reported technical problems, data loss or change in diagnosis as some of the reasons for participants to lose interest in recording their EMAs. The TYT users were not participants of a study, so we did not collect explicit feedback from them. However, our results from the analysis of the EMA themselves indicate that the responses of the users to some of the EMA items during the first two, three and four days of continuous interaction are predictive of whether the user will return after stopping the interaction or not: item q5 refers to arousal, a mediator of tinnitus distress^25^, item q1 refers to the perception of tinnitus at the moment of interaction, and item q6 refers to stress. This agrees partially with the findings of Courvoisier et al.^62^, who found a small but still significant correlation between compliance of tinnitus patients and their mood, as recorded in EMAs.

The results of the analysis of the TSCHQ questionnaire indicate a higher likelihood of adherence (${\textit{Return}}=\text {Yes}$) among older female users (aged 67 and 68). This agrees with the finding that female participants show a higher level of compliance to an EMA monitoring protocol^63^. However, it must be stressed that the female subpopulation in our data was very small.

The results of the analysis of the MiniTF questionnaire indicate no associations between adherence and condition at registration; an increased likelihood of adherence occurs both among users with worries about their health and among users with a more positive attitude at the moment of registration. Stratification of users on MiniTF scores does not show differences between users who return after an interruption and those that do not, except that there are substantially more of the former than the latter. Hence, we found no indication that user adherence can be predicted *before* the start of interaction with the app.

A threat to validity of our results is that the thresholds associated with adherence are ad hoc. Moreover, the number of users who interact only for one day with the mHealth app is very large and influences the model quality. Further, an independently identified software bug (different scales of Android and iOS) in the recording of EMA items q4 (mood) and q5 (arousal) may have suppressed the contribution of these two items to the prediction of adherence. The rapid deterioration of interaction duration and the heterogeneous patterns of interaction continuity indicate that the very first days of interaction with the app are decisive for user adherence. In the investigated version of TYT, the recording of EMA is a user activity that does not trigger any feedback. Different forms of feedback, e.g., praise in response to achievements or pointers to information sources, may have led to higher adherence.

Although we found no indication that adherence can be predicted before interaction with the app starts, we found that the mood of the users in the first few days can predict further interaction and that adherence deteriorates quickly. Hence, designers of smartphone-based EMA should consider ways of sustaining and stimulating the participant-app-interaction from the very beginning. Services (e.g., personalized feedback), entertainment (e.g., gamification) or social features are ways of stimulating interactions^64–66^. For example, within the Join Action CHRODIS-PLUS, the effects of tips for participants of a study were investigated using a follow-up version of TYT in the limited setting of a pilot study.

Being *momentary* assessments, EMA can deliver more detailed information on a study participant’s condition and at much shorter intervals than is possible during the interim visits performed regularly in a clinical study. Our results show that this benefit comes at a cost though, since clinical study designers must also put measures in place to sustain adherence during the whole time of the study. This demands a tighter integration of the clinical study design with the design of the participant-app-interaction. For example, in the recently started Horizon 2020 project UNITI on the “Unification of treatments and Interventions for Tinnitus patients”, EMA-based monitoring of participants’ condition is planned to be conducted as part of a multi-armed randomized clinical trial, whereupon adherence towards the app will be promoted through educational services.

## Supplementary Information

Supplementary Information.
