# Supplementary material for: Understanding adherence to the recording of ecological momentary assessments in the example of tinnitus monitoring
Source: Sci Rep. 2020 Dec 31;10:22459. doi: 10.1038/s41598-020-79527-0 (PMC7775469; doi:10.1038/s41598-020-79527-0)
Supplement: Supplementary file 1 — Supplementary Information. [file 41598_2020_79527_MOESM1_ESM.pdf]

# – Supplementary Material –

Understanding Adherence to the Recording of Ecological Momentary Assessments on the Example of Tinnitus Monitoring

Miro Schleicher[1,*], Vishnu Unnikrishnan[1], Jorge Simoes[2], Patrick Neff[2,3], Thomas Probst[4], Rüdiger Pryss[5], Winfried Schlee[2], and Myra Spiliopoulou[1]

[1]Institute of Technical and Business Information Systems, Otto-von-Guericke-University Magdeburg, Germany
[2]Department of Psychiatry and Psychotherapy of Regensburg University, Regensburg, Germany
[3]University Research Priority Program 'Dynamics of Healthy Aging', University of Zurich, Zurich, Switzerland
[4]Department for Psychotherapy and Biopsychosocial Health, Danube University Krems, Austria
[5]Institute of Clinical Epidemiology and Biometry, University of Würzburg, Würzburg, Germany
[*]Contact author: miro.schleicher@ovgu.de

Supplementary A: Translation of the MiniTF questionnaire items

The items of the MiniTF are depicted on Table 1, where we used a translation by Probst et al[1]. The answers to the questionnaire items are True (2 points), Partially True (1 point), Not True (0 points). MiniTF is used to derive a global score, ranging between 0 and 24 points. Higher values indicate higher severity (distress). Scores below 7 are of no pathological relevance.

Item	Translation of the German Question
tf1	I am aware of the noises from the moment I get up to the moment I sleep
tf2	Because of the noises I worry that there is something seriously wrong with my body
tf3	If the noises continue my life will not be worth living
tf4	I am more irritable with my family and friends because of the noises
tf5	I worry that the noises might damage my physical health
tf6	I find it harder to relax because of the noises
tf7	My noises are often so bad that I cannot ignore them
tf8	It takes me longer to get to sleep because of the noises
tf9	I am more liable to feel low because of the noises
tf10	I often think about whether the noises will ever go away
tf11	I am a victim of my noises
tf12	The noises have affected my concentration

Table 1. The translated items of the German MiniTF questionnaire of Hiller et al[2]; translation by Probst et al[1]

Supplementary B: Classification results on time series data of D[1292:852]

On Table 2 we see the accuracy achieved by each of the first 6 out of 13 algorithms for each EMA variable; on Table 3 we see the accuracy values for the remaining 7 algorithms. By taking apart the accuracy values for the individual variables, we can assess their contribution to separation between the two classes.

Algorithm	EMA item	value of *FirstDays*									
		1	2	3	4	5	6	7	8	9	10 or more
	q1	-	-	0.71	0.61	0.65	0.92	0.92	0.75	0.94	0.36
	q2	-	-	0.61	0.67	0.62	0.5	0.92	0.92	0.95	0.61
	q3	-	-	0.53	0.52	0.76	0.92	0.92	0.92	0.94	0.56
**ST**	q4	-	-	0.65	0.55	0.72	0.67	0.92	0.92	0.95	0.5
	q5	-	-	0.62	0.83	0.71	0.92	0.92	0.83	0.89	0.58
	q6	-	-	0.68	0.59	0.47	0.92	0.92	0.92	0.89	0.55
	q7	-	-	0.61	0.68	0.75	0.92	0.92	0.92	0.94	0.59
	q1	0.84	0.61	0.61	0.74	0.71	0.92	0.92	0.92	0.94	0.55
	q2	0.69	0.58	0.65	0.5	0.69	0.58	0.92	0.92	0.95	0.48
	q3	0.63	0.55	0.6	0.48	0.71	0.83	0.92	0.83	0.83	0.5
**TSF**	q4	0.77	0.52	0.48	0.64	0.78	0.83	0.92	0.92	0.95	0.5
	q5	0.76	0.61	0.69	0.61	0.76	0.92	0.92	0.92	0.95	0.55
	q6	0.68	0.54	0.54	0.55	0.71	0.58	0.92	0.83	0.94	0.48
	q7	0.64	0.6	0.61	0.55	0.56	0.75	0.92	0.92	0.78	0.56
	q1	-	0.54	0.52	0.74	0.82	0.92	0.92	0.92	0.89	0.55
	q2	-	0.58	0.68	0.5	0.69	0.75	0.92	0.92	0.89	0.64
	q3	-	0.47	0.57	0.39	0.71	0.92	0.92	0.83	0.83	0.5
**EE**	q4	-	0.48	0.42	0.59	0.72	0.67	0.92	0.92	0.95	0.53
	q5	-	0.66	0.62	0.61	0.71	0.92	0.92	0.92	0.89	0.48
	q6	-	0.54	0.43	0.68	0.76	0.75	0.92	0.83	0.94	0.48
	q7	-	0.45	0.58	0.64	0.69	0.75	0.92	0.92	0.78	0.53
	q1	0.84	0.54	0.48	0.74	0.65	0.92	0.92	0.92	0.89	0.48
	q2	0.73	0.58	0.65	0.5	0.75	0.92	0.92	0.92	0.84	0.61
	q3	0.73	0.48	0.57	0.39	0.82	0.92	0.92	0.83	0.83	0.44
**MSM**	q4	0.79	0.48	0.48	0.59	0.67	0.58	0.92	0.92	0.95	0.5
	q5	0.76	0.63	0.59	0.52	0.65	0.85	0.92	0.92	0.95	0.45
	q6	0.72	0.56	0.5	0.68	0.76	0.75	0.92	0.83	0.89	0.42
	q7	0.73	0.45	0.55	0.64	0.69	0.75	0.92	0.92	0.72	0.5
	q1	0.84	0.61	0.61	0.7	0.82	0.92	0.92	0.92	0.89	0.58
	q2	0.73	0.56	0.65	0.5	0.75	0.83	0.92	0.92	0.89	0.52
	q3	0.73	0.5	0.57	0.39	0.76	0.92	0.92	0.83	0.78	0.5
$\mathbf{CID_{DTW}}$	q4	0.79	0.53	0.55	0.59	0.72	0.67	0.92	0.83	0.95	0.56
	q5	0.76	0.63	0.59	0.61	0.71	0.85	0.92	0.83	0.89	0.58
	q6	0.72	0.54	0.54	0.68	0.76	0.67	0.92	0.83	0.89	0.35
	q7	0.73	0.44	0.55	0.59	0.62	0.67	0.92	0.92	0.72	0.53
	q1	0.84	0.62	0.65	0.78	0.65	0.92	0.92	0.92	0.89	0.55
	q2	0.73	0.61	0.68	0.5	0.69	0.75	0.92	0.92	0.89	0.67
	q3	0.73	0.5	0.57	0.39	0.65	0.92	0.92	0.83	0.83	0.47
$\mathbf{DTD_C}$	q4	0.79	0.53	0.58	0.59	0.67	0.58	0.92	0.92	0.95	0.53
	q5	0.76	0.61	0.62	0.61	0.65	0.92	0.92	0.83	0.79	0.48
	q6	0.72	0.54	0.43	0.64	0.76	0.75	0.92	0.83	0.94	0.48
	q7	0.73	0.44	0.58	0.64	0.69	0.67	0.92	0.92	0.72	0.56

Table 2. Accuracy (Return=Yes/No) of each time series classifier in dependence of *FirstDays* value (columns) and EMA item (row), for a horizon of observations of 30 days and a cut-off $\tau = 9$ days - first 6 of the classification algorithms

Algorithm	EMA item	value of *FirstDays*									
		1	2	3	4	5	6	7	8	9	10 or more
$\mathbf{DD_{DTW}}$	q1	0.84	0.61	0.65	0.74	0.71	0.92	0.92	0.92	0.89	0.52
	q2	0.81	0.56	0.65	0.67	0.69	0.83	0.92	0.92	0.89	0.58
	q3	0.81	0.48	0.53	0.39	0.76	0.92	0.92	0.83	0.94	0.47
	q4	0.82	0.47	0.58	0.59	0.67	0.58	0.92	0.83	0.9	0.5
	q5	0.81	0.61	0.62	0.61	0.71	0.85	0.92	0.83	0.95	0.55
	q6	0.8	0.52	0.43	0.68	0.53	0.92	0.92	0.92	0.94	0.35
	q7	0.81	0.44	0.55	0.64	0.69	0.67	0.92	0.92	0.72	0.47
**DTW**	q1	0.84	0.54	0.48	0.7	0.76	0.92	0.92	0.92	0.89	0.48
	q2	0.73	0.61	0.68	0.5	0.69	0.75	0.92	0.92	0.89	0.67
	q3	0.73	0.47	0.57	0.39	0.65	0.92	0.92	0.83	0.83	0.47
	q4	0.79	0.48	0.45	0.59	0.67	0.58	0.92	0.83	0.95	0.53
	q5	0.76	0.63	0.62	0.61	0.65	0.92	0.92	0.83	0.95	0.55
	q6	0.72	0.54	0.43	0.68	0.76	0.75	0.92	0.83	0.94	0.48
	q7	0.73	0.44	0.58	0.64	0.69	0.67	0.92	0.92	0.72	0.56
**RotF**	q1	0.84	0.52	0.71	0.7	0.71	0.92	0.92	0.92	0.94	0.52
	q2	0.81	0.53	0.61	0.71	0.75	0.92	0.92	0.92	0.95	0.55
	q3	0.81	0.53	0.63	0.7	0.76	0.92	0.92	0.92	0.94	0.59
	q4	0.82	0.6	0.52	0.68	0.78	0.92	0.92	0.92	0.95	0.53
	q5	0.81	0.58	0.69	0.65	0.76	0.92	0.92	0.92	0.95	0.61
	q6	0.8	0.56	0.57	0.68	0.76	0.92	0.92	0.92	0.94	0.58
	q7	0.81	0.52	0.68	0.68	0.75	0.92	0.92	0.92	0.94	0.59
**RandF**	q1	0.84	0.59	0.65	0.74	0.76	0.92	0.92	0.92	0.94	0.55
	q2	0.81	0.54	0.61	0.71	0.75	0.92	0.92	0.92	0.95	0.55
	q3	0.83	0.55	0.63	0.7	0.76	0.92	0.92	0.92	0.94	0.59
	q4	0.82	0.55	0.48	0.68	0.78	0.92	0.92	0.92	0.95	0.53
	q5	0.76	0.58	0.52	0.57	0.71	0.92	0.92	0.92	0.95	0.55
	q6	0.77	0.57	0.57	0.68	0.76	0.92	0.92	0.92	0.94	0.58
	q7	0.81	0.55	0.65	0.68	0.75	0.92	0.92	0.92	0.94	0.59
**C45**	q1	0.84	0.52	0.61	0.7	0.76	0.92	0.92	0.92	0.94	0.55
	q2	0.81	0.54	0.61	0.71	0.75	0.92	0.92	0.92	0.95	0.58
	q3	0.81	0.53	0.63	0.7	0.76	0.92	0.92	0.92	0.94	0.59
	q4	0.82	0.53	0.61	0.68	0.78	0.92	0.92	0.92	0.95	0.56
	q5	0.81	0.52	0.59	0.7	0.76	0.92	0.92	0.92	0.95	0.58
	q6	0.8	0.52	0.61	0.68	0.76	0.92	0.92	0.92	0.94	0.58
	q7	0.81	0.53	0.61	0.68	0.75	0.92	0.92	0.92	0.94	0.59
**NB**	q1	0.84	0.57	0.61	0.65	0.65	0.75	0.75	0.83	0.72	0.55
	q2	0.81	0.54	0.68	0.33	0.44	0.17	0.17	0.08	0.11	0.48
	q3	0.81	0.55	0.63	0.3	0.53	0.25	0.08	0.25	0	0.47
	q4	0.82	0.47	0.55	0.68	0.83	0.5	0.75	0.58	0.7	0.66
	q5	0.81	0.55	0.69	0.74	0.53	0.62	0.75	0.58	0.74	0.58
	q6	0.8	0.57	0.57	0.41	0.24	0.25	0.08	0.08	0.06	0.39
	q7	0.81	0.55	0.71	0.36	0.38	0.17	0.23	0.08	0.06	0.41
**ED**	q1	0.84	0.62	0.68	0.78	0.65	0.92	0.92	0.83	0.83	0.52
	q2	0.73	0.61	0.68	0.5	0.69	0.75	0.92	0.92	0.84	0.67
	q3	0.73	0.48	0.57	0.39	0.65	0.92	0.92	0.83	0.83	0.47
	q4	0.79	0.53	0.58	0.64	0.78	0.42	0.83	0.92	0.95	0.56
	q5	0.76	0.63	0.62	0.57	0.65	0.92	0.92	0.83	0.84	0.52
	q6	0.72	0.54	0.43	0.64	0.76	0.75	0.92	0.83	0.94	0.55
	q7	0.73	0.44	0.61	0.64	0.69	0.67	0.92	0.92	0.78	0.5

Table 3. Accuracy (Return=Yes/No) of each time series classifier in dependence of *FirstDays* value (columns) and EMA item (row), for a horizon of observations of 30 days and a cut-off $\tau_{early} = 9$ days - last 7 of the classification algorithms

References

1. Probst, T., Pryss, R., Langguth, B. & Schlee, W. Emotion dynamics and tinnitus: Daily life data from the ”trackyourtinnitus” application. *Sci. reports* **6** (2016).
2. Hiller, W. & Goebel, G. Rapid assessment of tinnitus-related psychological distress using the mini-tq. *Int. J. Audiol.* **43**, 600–604 (2004).
